# Agrowaste‐Derived Liquid Organic Fertilizer and Temperature Stabilization in Vertical Aeroponics for Crop Production

**DOI:** 10.1002/pei3.70153

**Published:** 2026-04-27

**Authors:** Litun Ahmed Labib, Swagata Ahmed, Muhammad Abdul Malek, Md. Fakhrul Hasan

**Affiliations:** ^1^ Department of Horticulture Patuakhali Science and Technology University Patuakhali Bangladesh; ^2^ Department of Horticulture Gazipur Agricultural University Gazipur Bangladesh

**Keywords:** agrowaste, kangkong ( *Ipomoea aquatica* ), liquid organic fertilizer, temperature stabilization, vertical aeroponics

## Abstract

Sustainability challenges in hydroponic and aeroponic systems are primarily due to the limited use of liquid organic fertilizers (LOFs) and the suboptimal temperature conditions for plant growth. This study aims to address these issues by incorporating agrowaste‐derived LOFs as an alternative nutrient source and utilizing a clay‐dominant soil layer in the vertical aeroponic system to stabilize nutrient solution temperatures. The goal is to optimize both the productivity and efficiency of LOFs. Eight LOFs were developed using mustard oil cake, sugarcane, and kangkong leaves, and assessed on various parameters. Among these, formulations F6 and F4 exhibited optimal pH (4.50 and 5.0), electrical conductivity (18.20 and 18.10 dS/m), elevated levels of nitrogen (0.19% in both), phosphorus (0.017% and 0.018%), and potassium (1.10% in both). To address temperature fluctuations in the nutrient solution, a 1.5‐in. layer of silty clay soil effectively stabilized nutrient temperatures, reducing variation from 30.90°C to 27.50°C in summer and from 22.63°C to 19.57°C in winter. Leafy vegetable Kangkong ( *Ipomoea aquatica* ) was used in this study, where various growth and nutritional parameters were evaluated over 45 days to assess the impacts of various treatments. Plants grown with liquid organic fertilizer F4 (treatment T4) achieved a yield of 484.80 g/unit plot, which was comparable to the non‐organic treatment T2 at 505.30 g/unit plot with similar nutritional quality. These findings demonstrate that agrowaste‐derived LOFs can serve as a sustainable alternative to synthetic fertilizers in aeroponic systems, offering both environmental and agronomic benefits.

## Graphical abstract

Agrowaste‐derived liquid organic fertilizers (LOFs) combined with a silty clay layer improved nutrient efficiency and stabilized root‐zone temperature in vertical aeroponics. Formulation F4 achieved yield (484.8 g/plot) comparable to non‐organic fertilizer (505.3 g) with similar nutritional quality, demonstrating a sustainable alternative for efficient crop production.

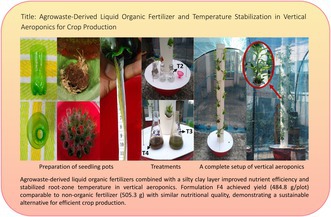

## 1 Introduction

The world population is predicted to reach an estimated 9.7 billion by 2050 and cities will be hosting about 80% of this population (Joshi et al. 2024). Currently, there are approximately 500 billion hectares of land that are designated for soil‐based farming globally, which constitutes about 38% of the total global land area (World Food and Agriculture 2025). Moreover, 80% of the global arable land is currently being used (Olsson et al. 2023). As the demand for food increases, there is a growing need to expand arable land and intensify farming practices, which will have significant impacts on global agriculture. To address this demand, vertical farming has emerged as a novel agricultural approach that involves large‐scale food production within high‐rise buildings, where environmental conditions and nutrient solutions are carefully controlled using hydroponics and advanced greenhouse technologies to promote rapid crop growth and efficient production (Pennisi et al. 2025; Sowmya et al. 2024; Van Gerrewey et al. 2021). As an advanced iteration of hydroponics, vertical aeroponics boasts simplicity and efficacy, distinguishing it as a cutting‐edge solution for modern agriculture. Its unique ability to be vertically implemented enables the optimization of space while ensuring cultivation free from pesticides and herbicides (Chu and Wan 2026; Garzón et al. 2023). This innovative method accelerates growth, enhances plant health, and increases yields by misting plant roots with water and nutrients, providing oxygen directly from the air and reducing resource use through automation, water‐nutrient recycling, and lower labor costs (Lawrance et al. 2025; Schmidt Rivera et al. 2023).

Despite its significant advantages, the primary drawback of this system is its dependence on chemical nutrients (Rajendran et al. 2024; Gumisiriza et al. 2023). These chemical nutrients are costly, not widely available, and require specialized technical expertise to prepare. In addition, the application of these chemical nutrients in hydroponic systems tends to cause a gradual increase in nitrate (NO_3_) accumulation, particularly in vegetable leaves (Park and Williams 2024; Gent 2003). For instance, Fang and Chung (2018) found that organically grown lettuce had 800–2000 ppm of nitrate, compared to 5000–5500 ppm with hydroponic chemical nutrients. Similarly, Williams and Nelson (2016) observed ‘Rex’ butterhead lettuce had 35–200 ppm with organic nutrients, versus 600–1100 ppm with hydroponic chemical nutrients. High nitrate accumulation poses various health risks by promoting the formation of toxic N‐nitrosamines (Kiani et al. 2022). Furthermore, non‐organically grown produce may contain various harmful chemical residues (Poulia et al. 2025).

On the contrary, there has been a notable increase in global demand for organic food over the past decade (Meshram et al. 2026). Consequently, organic vertical farming has emerged as an ideal solution to meet this rising demand and overcome the related challenges (Kumar et al. 2023). It plays a key role in developing technologies that optimize the interconnections between food, energy, water, and repurposed waste, offering potential benefits (Park and Williams 2024; Gomiero 2018; Barański et al. 2014). Moreover, liquid organic fertilizers for hydroponics produced from agricultural and industrial wastes through anaerobic digestion are becoming increasingly popular. These fertilizers are rich in essential macro and micronutrients, which are typically insoluble in water but are slowly converted into soluble forms for efficient absorption by plants and prolonged availability (Wang et al. 2025; Phibunwatthanawong and Riddech 2019).

Bangladesh, located in the northeastern part of South Asia, experiences a tropical monsoon climate with significant temperature fluctuations. In summer, temperatures frequently exceed 38°C, while winter temperatures can drop below 15°C (The Daily Star 2024a, 2024b). This variability makes it challenging to maintain a consistent temperature in the nutrient solution. During the hot summer months, high temperatures can cause nutrient concentrations to rise, disrupting the nutrient balance and hindering the plant's ability to absorb both water and nutrients (Hooks et al. 2022; Gent 2016; Nxawe et al. 2010). Conversely, the cold winter temperatures can damage plant roots and limit nutrient uptake, severely affecting growth (Al‐Meselmani 2022; Thakulla et al. 2021). In traditional soil‐based farming, soil serves as a thermal barrier, protecting roots from excessive heat and cold injury. The composition of soil, particularly its clay content, plays a crucial role in enhancing its thermal insulation properties, where higher clay content offers enhanced thermal resistance (Petcu et al. 2023). Thus, the use of a high clay content soil layer of optimal thickness encasing the nutrient reservoir could serve as an effective strategy for mitigating temperature fluctuations in the nutrient solution.

Based on the available literature, no study has comprehensively addressed the issue of minimizing nutrient solution temperature fluctuations or the application of liquid organic fertilizers in vertical aeroponic systems. These drawbacks make the system vulnerable to areas where temperature fluctuation is high, hindering its productivity and causing poor quality of food. Therefore, the objectives of this study were to develop a climate‐resilient vertical aeroponic system by incorporating a clay‐dominant soil layer in the nutrient reservoir to stabilize nutrient solution temperature, to produce liquid organic fertilizers from agrowaste, and to evaluate their performance within the system. It is hypothesized that (i) liquid organic fertilizers derived from agrowaste can provide sufficient nutrients to support plant growth comparable to conventional non‐organic (synthetic) fertilizers; (ii) the incorporation of a clay‐dominant soil layer around the nutrient reservoir will significantly reduce temperature fluctuations and improve root‐zone conditions; and (iii) the combined application of optimized liquid organic fertilizers and temperature stabilization will enhance crop productivity and nutritional quality in vertical aeroponic systems.

## 2 Materials and Methods

### 2.1 Preparation of Liquid Organic Fertilizers

Liquid organic fertilizers were developed using organic agrowastes and agricultural by‐products, including mustard oil cake, sugarcane leaves, kangkong leaves, and water. Kangkong and sugarcane leaves were freshly harvested and cut into 1.5–2.0 cm pieces. Mustard oil cake was purchased from the local market and pulverized with a grinder. Distilled water was used as a water source. The different liquid organic fertilizer formulations were prepared by mixing the substrates in various proportions and storing the mixtures in sealed plastic bottles. Anaerobic digestion was carried out at room temperature for 30 days with stirring every 3‐day intervals using a glass rod. Based on preliminary studies employing a stepwise ratio adjustment approach, eight optimized formulations were selected and prepared: F1 = (mustard oil cake + sugarcane leaf + water, 1:1:0.25 v:w:v), F2 = (mustard oil cake + sugarcane leaf + water, 1.5:1:0.25 v:w:v), F3 = (mustard oil cake + sugarcane leaf + water, 1.5:0.25:0.25 v:w:v), F4 = (mustard oil cake + sugarcane leaf + water, 2:1:0.25 v:w:v), F5 = (mustard oil cake + kangkong leaf + water, 1:1:0.25 v:w:v), F6 = (mustard oil cake + kangkong leaf + water, 1.5:1:0.25 v:w:v), F7 = (mustard oil cake + kangkong leaf + water, 1.5:0.25:0.25 v:w:v), F8 = (mustard oil cake + kangkong leaf + water, 2:1:0.25 v:w:v). Each treatment was prepared in triplicate (*n* = 3). The formulations were sterilized using an autoclave (Figure S1).

### 2.2 Assessment of Liquid Organic Fertilizers

The efficacy of liquid organic fertilizers was evaluated across various parameters employing a range of analytical methods, with data collection performed at 15 and 30 days after the anaerobic digestion. pH was measured using a glass electrode pH meter (GLP 21, Crison, Barcelona, ECC), while electrical conductivity (EC) was determined using a glass electrode EC meter and expressed in deciSiemens per meter (dS/m). For essential plant nutrients, total nitrogen (N) content was analyzed using the Kjeldahl method, total phosphorus (P) was determined by the wet digestion spectrophotometric method at 420 nm, and total potassium (K) was measured using a flame photometer.

### 2.3 Temperature Stabilization of Nutrient Solution

To minimize fluctuations in nutrient solution temperature and maintain stability, a method was employed using two plastic bowls of 15 and 20 L instead of a single bowl for the nutrient reservoir (Figure S2). The 15‐l bowl was positioned inside the 20‐l bowl, resulting in a gap between them because of the size difference. The gap was filled with soil at three specific thicknesses (0.5, 1.0, and 1.5 in.) to serve as a thermal barrier. Silty clay soil was used in the experiment considering its unique characteristics, including a brownish‐gray color, soft creamy texture, and organic‐rich composition with over 50% clay content, as higher clay content provides enhanced thermal resistance (Petcu et al. 2023; Yincan 2017). To ensure uniform thickness, a pre‐marked 2‐in. stainless steel stick was inserted into multiple sites of the soil layer and adjusted to achieve the desired thickness. The temperature of the nutrients and the surrounding environment was measured using an HTC‐2 Digital thermo‐hygrometer. The study was conducted in both summer (June) and winter (January) across three consecutive days. Temperature measurements were carried out at six specific times of each day (12 PM, 4 PM, 8 PM, 12 AM, 4 AM, and 8 AM), and the mean of these readings was calculated for analysis.

### 2.4 Planting Material and Preparation of Vertical Aeroponics Setup

Kangkong ( *Ipomoea aquatica* ) was used in this study. The vertical aeroponic structures were constructed using 6.5 ft long and 6.0‐in. diameter hard white plastic pipe, along with two plastic bowls of 15 and 20 L, a 12 V mini electric pump connected to a 7 ft long thin (4 cm) flexible plastic pipe, a sprinkler, hardboard covers, and ropes. Each vertical pipe contained four equidistant vertical rows with five planting holes per row (20 holes per unit). Seedling holders (≈5.5 in length) were fabricated from recycled plastic bottles, serving as an effective way to repurpose materials and cut down on waste. To construct the base of the aeroponics system, a 15‐l bowl was placed inside a 20‐l bowl, and the gap was filled with a 1.5‐in.‐thick layer of silty clay soil (Section 3.2). A 12 V mini electric pump was placed in the bowl and connected to a high‐pressure sprinkler at the top of the structure via a flexible pipe. Seedling pots were prepared by filling each pot with one‐quarter coco husk and coco peat, and 2–3 kangkong seeds were placed in each pot. After 10 days of emergence, seedling pots were transplanted into the aeroponic systems by keeping one healthy plant in each. After transplantation, the predetermined treatments (Section 2.5) were applied in aeroponic systems and the seedlings received a light misting of nutrients for 1 h. A circulating floating technique was used, and the nutrient solution circulation was controlled by a timer (Nearpow CECOMINOD036912) set to run for 15 min every hour, followed by 45 min of rest until harvest (Figure S3).

### 2.5 Experimental Design and Treatments

The experiment was conducted in a net house under intensive care, where neither weeding nor disease and pest control were required (Figure S4). The net house was meticulously maintained throughout the duration of the experiment. The study consisted of four treatments arranged in a completely randomized design (CRD) with four replications (*n* = 4) per treatment: T1 = Pot culture (negative control), T2 = Non‐organic/Chemical nutrients (positive control), T3 = Liquid organic fertilizer formulation F6 (mustard oil cake + kangkong leaf + water, in a ratio of 1.5:1:0.25 v:w:v), T4 = Liquid organic fertilizer formulation F4 (mustard oil cake + sugarcane leaf + water, in a ratio of 2:1:0.25 v:w:v). These two most promising liquid organic fertilizer formulations (F4 and F6) were selected based on pH, EC, and the availability of essential nutrients (Section 3.1), and were subsequently diluted with tap water at a 1:4 ratio for application in aeroponic systems, with applications made at 7‐day intervals. In pot culture, loamy soil was used, and fertilizers were applied according to the guidelines specified in Ahmmed et al. (2018). Chemical nutrients (stock solutions A and B) were prepared according to the recommendation of the Bangladesh Agricultural Research Council (2023) and were used at a ratio of 1:1:100 with tap water (Table S1). The pH of the nutrient solution in the aeroponic system was kept within the range of 6.5–7.0 using nitric acid (HNO_3_) or potassium hydroxide (KOH), and EC was maintained between 1.8–2.0 dS/m throughout the entire production period (Endoh et al. 2024).

### 2.6 Data Collection, Assessment, and Statistical Analyses

Plant growth, yield attributes, and physicochemical properties were evaluated under different treatments. Data were recorded at 15, 30, and 45 days after transplanting (DAT). In pot culture, total yield was determined as the cumulative fresh weight of plants grown at 10–15 cm spacing within an equivalent ground area (75 ± 5 cm^2^) to that used in the vertical aeroponic system. Physicochemical properties of kangkong were analyzed following the methodologies described by Ahmed et al. (2024) and Labib et al. (2025). Collected data were analyzed using one‐way analysis of variance (ANOVA) to evaluate the significance of the treatments. Mean values were separated using Fisher's Least Significant Difference (LSD) at the 5% level of significance (*p* ≤ 0.05). Results are presented as mean ± standard deviation (SD). All statistical analyses and graphical visualizations were performed using R software (v4.4.1).

## 3 Results and Discussion

### 3.1 Liquid Organic Fertilizers

#### 3.1.1 pH of Liquid Organic Fertilizers

**FIGURE 1:**
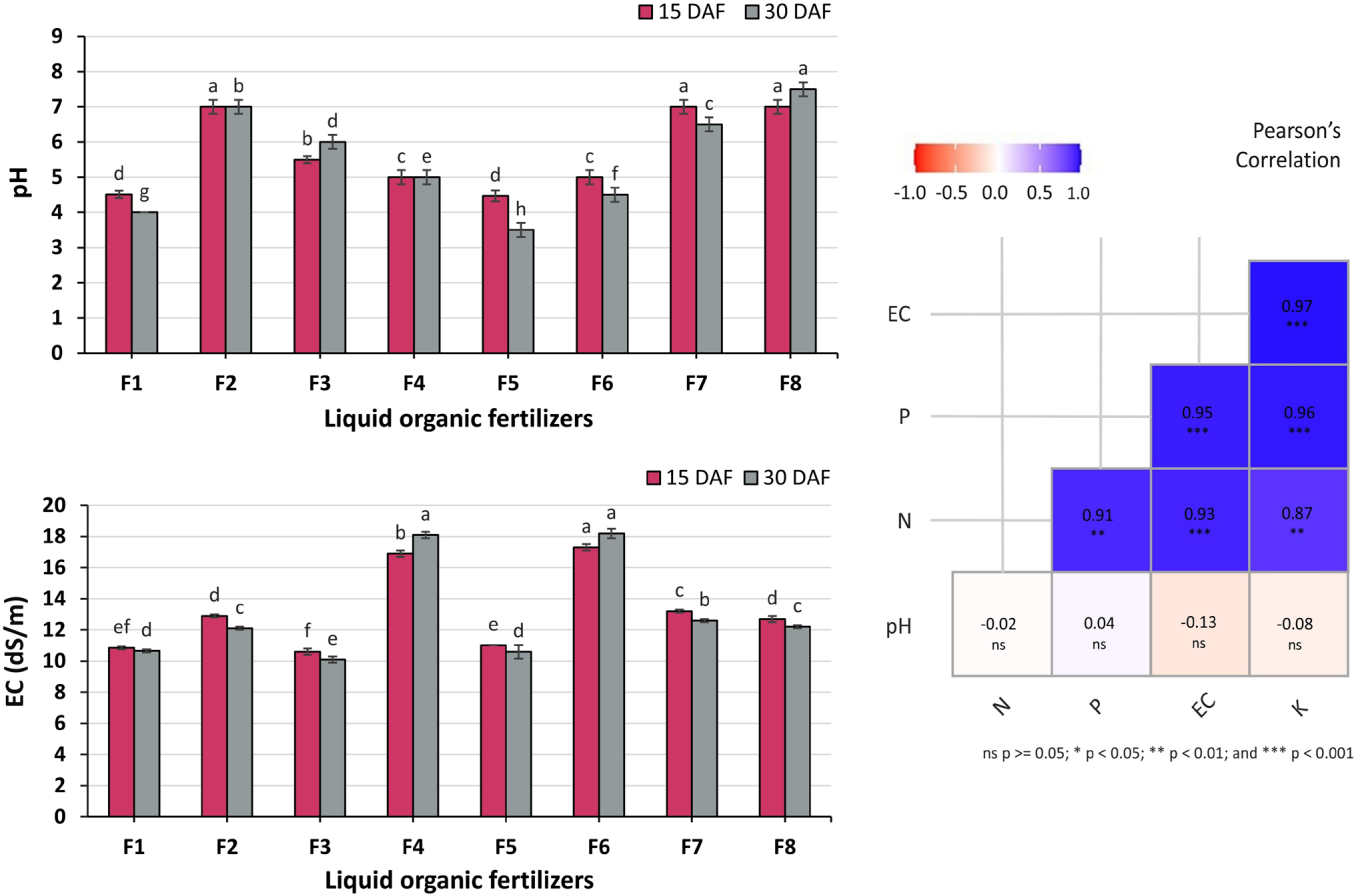
pH and electrical conductivity (EC) of various liquid organic fertilizers, along with Pearson correlations among key variables. Different lowercase letters indicate significant differences among treatments (*p* ≤ 0.05). Vertical bars represent the standard error of the mean (*n* = 3).

The pH of liquid organic fertilizers was measured at 15 and 30 days after fermentation (DAF). Significant differences (*p* ≤ 0.05) were observed among the formulations at both intervals (Figure 1). At 15 DAF, the pH values ranged from 4.47 to 7.00. The F2 formulation had the highest pH of 7.00, followed by F7 and F8. Formulations F3, F4, and F6 exhibited pH values of 5.50, 5.00, and 5.00. The lowest pH of 4.47 was recorded in formulation F5. After 30 DAF, pH values spanned from 3.50 to 7.50. Here, the F8 formulation showcased the highest pH of 7.50, followed by F2 at 7.00 and F7 at 6.50. Subsequently, a decline in pH was observed, where F3 reported a pH of 6.00, F4 at 5.00, and F6 at 4.50. The F5 formulation retained the lowest pH at 3.50. The acidic pH values were a result of microorganisms converting the sugars in the substrate into lactic acid or acetic acid. High‐quality liquid organic fertilizers are typically expected to maintain a pH level below 5.5 (Iqbal et al. 2024; Phibunwatthanawong and Riddech 2019; NARO 2012), and the results demonstrated that F6 and F4 pose ideal pH (5.0 and 4.5) for liquid organic fertilizer. Moreover, plant nutrient availability depends on pH. If the pH is too high (alkaline) or too low (acidic), essential nutrients may become less soluble or precipitate (Barrow and Hartemink 2023). Therefore, the pH of liquid organic fertilizers is an essential parameter influencing their effectiveness and compatibility with nutrient solutions and plant growth.

#### 3.1.2 EC of Liquid Organic Fertilizers

Electrical conductivity (EC) of liquid organic fertilizers revealed significant differences (*p* ≤ 0.05) among the formulations (Figure 1). After 15 days after fermentation (DAF), the F6 formulation exhibited the highest EC at 17.30 dS/m, followed by the F4 formulation at 16.90 dS/m. In contrast, the F3 formulation displayed the lowest EC at 10.60 dS/m. Similarly, at 30 DAF, the F6 formulation retained its position with the highest EC of 18.20 dS/m, which was statistically similar to the EC of the F4 formulation at 18.10 dS/m. Once again, the F3 formulation had the lowest EC at 10.10 dS/m. The elevated EC values observed in these formulations may be associated with the high content of natural substrates. The increase in EC was driven by microbial activity that mineralized nutrients from organic fertilizers, influenced by factors such as growing media, temperature, moisture, C/N ratio, oxygen availability, and pH (Sradnick and Feller 2020; Meeboon et al. 2022). EC indicates the concentration of nutrients in a solution, with higher values meaning more nutrients and excessively high levels signaling salt buildup (Ding et al. 2018). Maintaining optimal EC ensures plants receive a balanced nutrient mix that promotes healthy growth and strong roots (Nguyen et al. 2021). These findings align with the recommendations of Phibunwatthanawong and Riddech (2019), which suggest that optimal liquid organic fertilizers should ideally possess an EC value of less than 20 dS/m to ensure suitability for agricultural use. Based on this criterion, F6 and F4 exhibit strong potential as high‐quality liquid organic fertilizers, as their EC values remained within the desired range throughout the fermentation process.

#### 3.1.3 Essential Nutrient Content of Liquid Organic Fertilizers

The analysis of nutrient composition in liquid organic fertilizers unveiled significant variations (*p* ≤ 0.001) in total nitrogen (N), available phosphorus (P), and exchangeable potassium (K) (Table 1). At 15 days after fermentation (DAF), formulations F6 and F4 exhibited the highest N percentages, at 0.22% and 0.21%, while the lowest value was observed in formulation F3 at 0.08%. Similarly, at 30 DAF, the N percentage remained higher in F6 and F4 at 0.19%, whereas F3 again showed the lowest value at 0.07%. Regarding P, the F4 and F6 formulations exhibited the highest percentages at 15 DAF, at 0.017% and 0.016%, while the lowest value was observed in F3 at 0.005%. This pattern continued at 30 DAF, with F4 and F6 maintaining the highest P percentages at 0.018% and 0.017%, whereas F3 retained the lowest value at 0.005%. In terms of K, formulations F4 and F6 exhibited the highest percentage at 1.09% at 15 DAF, while the lowest value was observed in F3 at 0.68%. This trend persisted at 30 DAF, where the maximum K content was again recorded in both F4 and F6 at 1.10% in both, whereas the lowest value was observed in F5 at 0.63%. During fermentation, the increase in total N content may be attributed to the activity of N‐fixing microorganisms (Zhou et al. 2022). Nitrogen is a key component of amino acids, proteins, and chlorophyll, the pigment essential for photosynthesis. It also interacts with phytohormones to regulate plant growth, enhance nutrient uptake, and coordinate developmental processes for optimal nutrition (Kiba et al. 2011). Similarly, the observed increase in P and K content may result from microbial decomposition of organic matter, whereby higher levels of organic material contribute to elevated P and K concentrations (Ratrinia et al. 2016). P is essential for ATP production, energy transfer, root development, flowering, fruiting, seed production, and the formation of DNA and RNA in plants (Khan et al. 2023). K aids in photosynthesis and the synthesis of proteins and carbohydrates, maintaining plant turgor. Gibberellin promotes cell division and enlargement, also enhancing turgor. This suggests a synergistic relationship between K and gibberellin (Hasanuzzaman et al. 2018). These macronutrients play an essential role in the growth, development, and overall health of plants in hydroponic systems. The F4 and F6 formulations, which consistently exhibited high concentrations of N, P, and K, are promising as nutrient‐rich liquid organic fertilizers. These findings are consistent with those of Phibunwatthanawong and Riddech (2019), Shaik et al. (2022), and Alneyadi et al. (2024), who also found that liquid organic fertilizers with similar N, P, and K concentrations are effective in enhancing plant growth and development.

**TABLE 1:** Essential nutrient content of liquid organic fertilizers: Nitrogen (N), Phosphorus (P), and Potassium (K).

LOF formulations	15 days after fermentation			30 days after fermentation		
	Total N (%)	Total P (%)	Total K (%)	Total N (%)	Total P (%)	Total K (%)
F1	0.11 c	0.008 c	0.72 d	0.09 cd	0.009 c	0.73 cd
F2	0.13 bc	0.010 bc	0.78 c	0.09 cd	0.010 bc	0.76 bc
F3	0.08 d	0.005 d	0.68 e	0.07 d	0.005 d	0.68 cd
F4	0.21 a	0.017 a	1.09 a	0.19 a	0.018 a	1.10 a
F5	0.09 d	0.009 bc	0.69 e	0.08 cd	0.005 d	0.63 d
F6	0.22 a	0.016 a	1.09 a	0.19 a	0.017 a	1.10 a
F7	0.12 bc	0.011 b	0.82 b	0.10 c	0.012 b	0.88 b
F8	0.14 b	0.009 bc	0.76 c	0.15 b	0.011 bc	0.74 cd
Level of sig.	***	***	***	***	***	***
CV (%)	9.00	11.12	2.20	11.02	10.66	8.73
LSD_0.05_	0.0215	0.0020	0.0316	0.0229	0.0020	0.1249

*Note:* Here, the different lowercase letters in a column indicate significant differences among different treatments (*p* ≤ 0.05), LOF = Liquid organic fertilizer. Significance level: ‘***’ *p* ≤ 0.001.

#### 3.1.4 Pearson Correlation Analysis of Liquid Organic Fertilizers

Pearson correlation analysis was conducted to assess the interrelationships between the studied variables (Figure 1). A highly significant (*p* ≤ 0.001) positive correlation (R^2^ = 0.97, 0.95, 0.93) was observed between EC and the nutrient parameters K, P, and N. These results indicate that as EC increases, the concentrations of these nutrients also increase. On the other hand, correlations between pH and N, P, K, EC were found to be statistically insignificant (*p* > 0.05, R^2^ = −0.02, 0.04, −0.08, −0.13), indicating that pH does not directly affect nutrient concentrations in liquid organic fertilizers. However, although pH may not directly influence nutrient concentrations, it is known to affect nutrient availability and can impact fertilizer efficiency (Barrow and Hartemink 2023). Furthermore, N exhibited significant (*p* ≤ 0.01) correlation coefficients with both P and K, with R^2^ values of 0.91 and 0.87. Additionally, a strong positive correlation was observed between P and K (R^2^ = 0.96). These findings suggest a tightly interconnected relationship between these essential nutrients, which could be crucial for optimizing fertilizer formulations. These findings emphasize the importance of maintaining optimal EC in aeroponic nutrient solutions to ensure adequate nutrient availability for plant growth. It provides valuable insights into the relationship between EC and nutrient dynamics in liquid organic fertilizers, which can help enhance fertilizer strategies for plant cultivation systems.

### 3.2 Nutrient Temperature Stabilization of Vertical Aeroponic System

**FIGURE 2:**
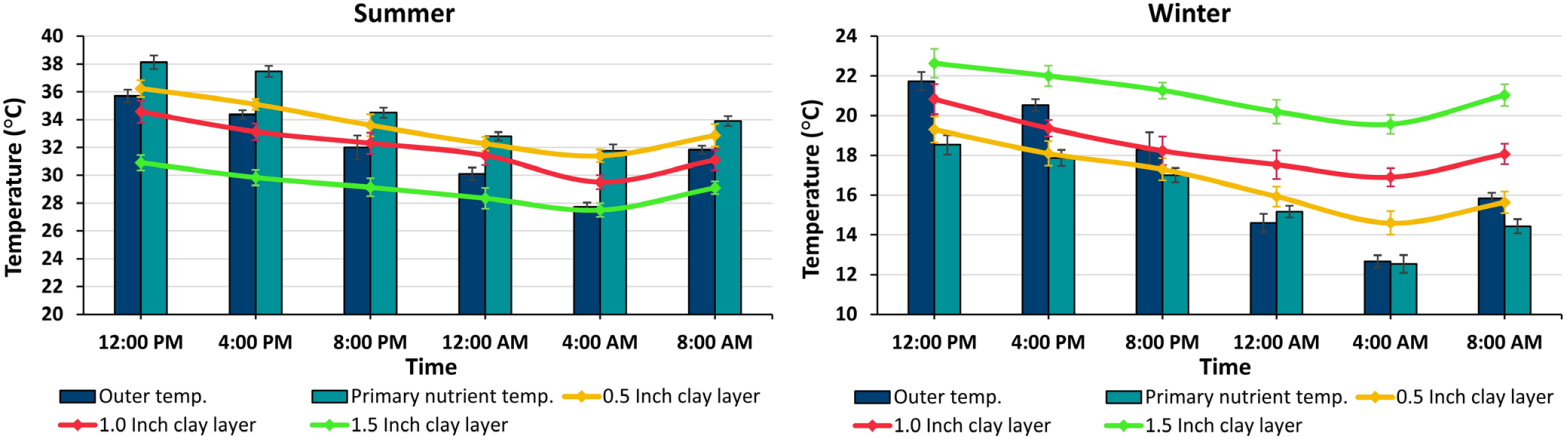
Effect of silty clay soil layer on temperature stabilization of nutrient solution in vertical aeroponic system. Vertical bars represent the standard error of the mean (*n* = 3).

The incorporation of silty clay soil layers of different thicknesses in the surroundings of the nutrient reservoir of the aeroponic system had a significant effect on the nutrient temperatures measured during both the summer (June) and winter (January) seasons (Figure 2). In summer, the external temperature varied between 35.70°C and 27.73°C, whereas the nutrient temperature in the absence of soil intervention was higher, ranging from 38.12°C to 31.77°C. However, the incorporation of the soil layer led to a notable reduction in nutrient temperatures. A 0.5‐in. layer reduced the nutrient temperature range from 36.23°C to 31.40°C, while a 1.0‐in. layer further decreased it to 34.57°C to 29.50°C. The most substantial decrease was observed with a 1.5‐in. soil layer, where temperatures ranged from 30.90°C to 27.50°C. This reduction indicates the efficacy of the soil layer in stabilizing temperatures, mitigating the adverse effects of external temperature fluctuations. In winter, the external temperature ranged from 21.73°C to 12.67°C, with nutrient temperatures in the untreated system falling between 18.53°C and 12.53°C. The application of the soil layer resulted in a noticeable increase in nutrient temperatures. A 0.5‐in. soil layer raised the nutrient temperature range from 19.30°C to 14.60°C, while a 1.0‐in. layer further increased it to 20.83°C to 16.90°C. The most significant increase was evident with a 1.5‐in. layer, where temperatures ranged from 22.63°C to 19.57°C. This rise suggests that the application of the soil layer surrounding the nutrient reservoir effectively prevented heat loss, thereby maintaining more optimal growing conditions during the colder winter months. These findings are consistent with the work of Levine et al. (2023) and He et al. (2013), who highlighted that a root zone temperature of 20°C to 34°C is vital for the growth of aeroponically and hydroponically grown lettuce. Temperatures exceeding this range lead to poor photosynthesis, reduced nitrogen uptake, and lower growth and yield. Moreover, Nisar et al. (2024) discovered that reducing the nutrient temperature during summer enhanced plant growth and yield by ensuring optimal nutrient concentrations. Similarly, Hayashi et al. (2024) demonstrated that increasing the root zone temperature during winter in hydroponic systems enhanced lettuce growth and yield by preventing root cold injury and improving nutrient uptake. In line with the hypothesis, these findings suggest that the 1.5‐in. silty clay soil layer demonstrates the most effective temperature stabilization across both summer and winter. This layer not only reduced temperature fluctuations but also decreased the day‐to‐night temperature variation by nearly 50%, promoting a more favorable growing environment. Additionally, the temperature of the nutrient solution can be adjusted by adding hot or cold water to the soil layer rather than directly introducing it into the nutrient solution, which could disrupt the nutrient balance.

### 3.3 Growth and Physicochemical Properties of Kangkong

#### 3.3.1 Number of Leaves Per Plant

**FIGURE 3:**
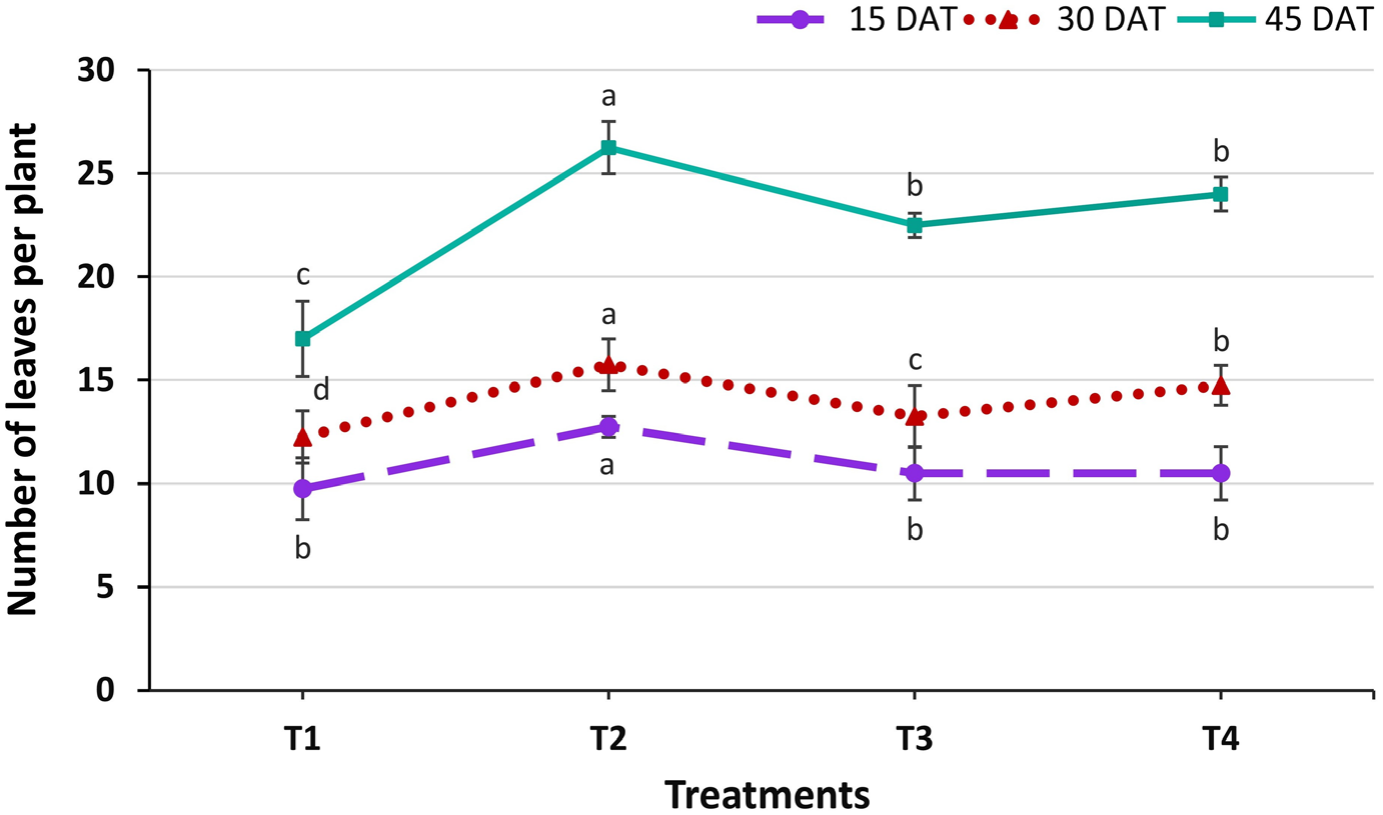
Effect of different treatments on the number of leaves per kangkong plant. Here, different lowercase letters indicate significant differences (*p* ≤ 0.05). Vertical bars represent the standard error of the mean (*n* = 4).

The number of leaves per kangkong plant was significantly (*p* ≤ 0.05) influenced by the different treatments at all growth stages (Figure 3). At 15 days after transplanting (DAT), the maximum number of leaves was observed in treatment T2 at 12.75, whereas the minimum was in T1 at 9.75, statistically similar to T3 and T4 treatments. At 30 DAT, T2 again produced the maximum leaf count at 15.75, followed by T4 at 14.75, whereas the minimum was recorded in T1 at 12.25. At 45 DAT, T2 maintained the highest leaf number at 26.25, followed by T4 at 24.00, while T1 consistently showed the lowest value at 17.00. The results indicate that the non‐organic treatment T2 produced the highest leaf number, whereas T1 (pot culture) recorded the lowest, likely due to enhanced growth under aeroponic conditions where roots have continuous access to oxygenated water and nutrients (Lawrance et al. 2025). Notably, the organic treatment T4 produced the second‐highest leaf number, indicating its effectiveness in supplying nutrients for sustained plant growth. These findings are consistent with previous studies by Alneyadi et al. (2024) and Ahmed et al. (2021), who reported improved leaf development in lettuce under organic nutrient management in hydroponic NFT systems.

#### 3.3.2 Length and Breadth of the Largest Leaf

**FIGURE 4:**
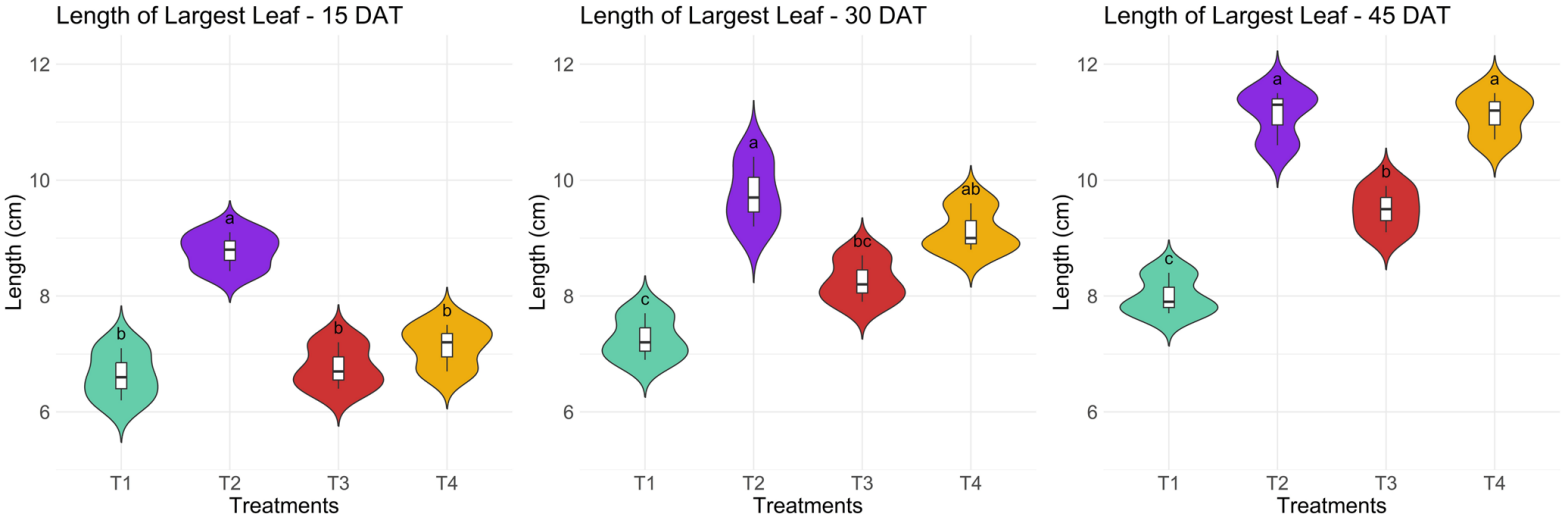
Effect of different treatments on the length of the largest leaf in kangkong. Here, different lowercase letters indicate significant differences (*p* ≤ 0.05). Vertical bars represent the standard error of the mean (*n* = 4).

**FIGURE 5:**
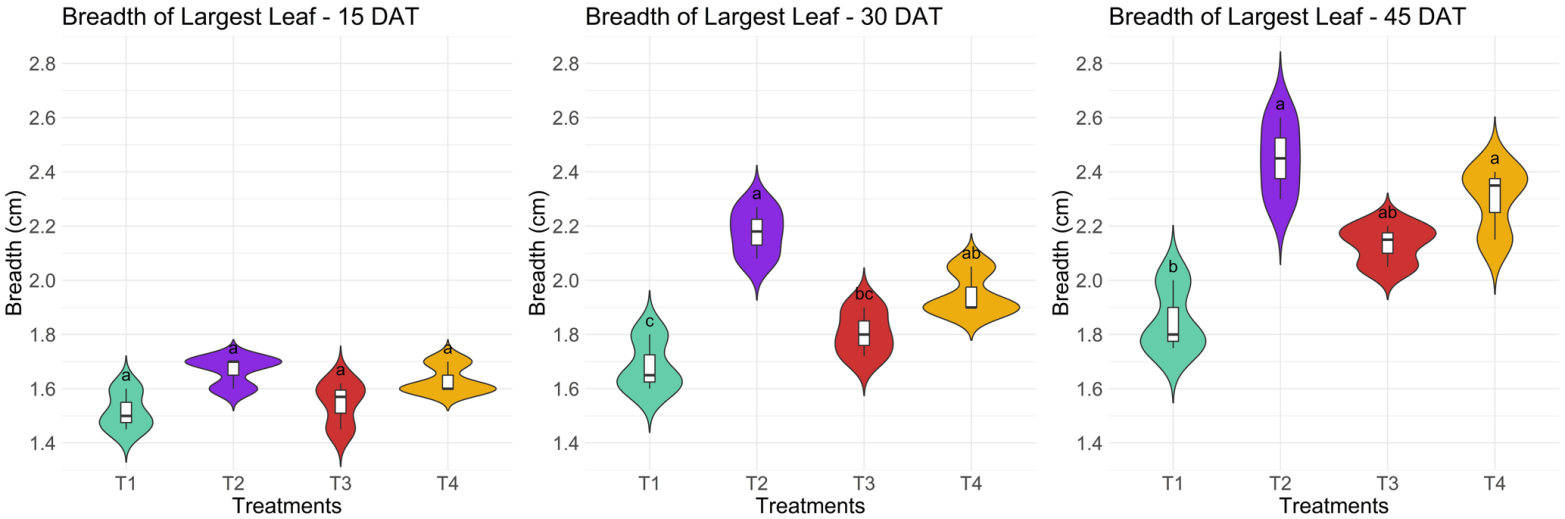
Effect of different treatments on the breadth of the largest leaf in kangkong. Here, different lowercase letters indicate significant differences (*p* ≤ 0.05). Vertical bars represent the standard error of the mean (*n* = 4).

The length and breadth of the largest leaf in kangkong were significantly (*p* ≤ 0.05) affected by different treatments at 15, 30, and 45 days after transplanting (DAT) (Figures 4 and 5). At 15 DAT, the longest leaf length was observed in treatment T2 at 8.78 cm, followed by T4 at 7.13 cm, T3 at 6.75 cm, and T1 at 6.63 cm. No significant differences were found in leaf breadth at this stage. At 30 DAT, T2 again produced the longest leaf at 9.78 cm, while T1 had the shortest at 7.25 cm. The widest leaf was found in T2 at 2.18 cm, followed by T4 at 1.95 cm, while T1 had the narrowest leaf at 1.68 cm. At 45 DAT, both T2 and T4 showed the longest leaves at 11.13 cm, whereas T1 had the shortest at 8.00 cm. The broadest leaf was recorded in T2 at 2.45 cm, followed by T4 at 2.30 cm, with the narrowest being in T1 at 1.85 cm. The results indicate that non‐organic treatment T2 consistently produced the longest and broadest leaves, while the lowest performance was observed in the pot culture treatment T1. On the contrary, organic treatment T4 did not exhibit any significant difference in all measured growth parameters compared to the results obtained using chemical nutrients.

#### 3.3.3 Plant Height and Root Length

**FIGURE 6:**
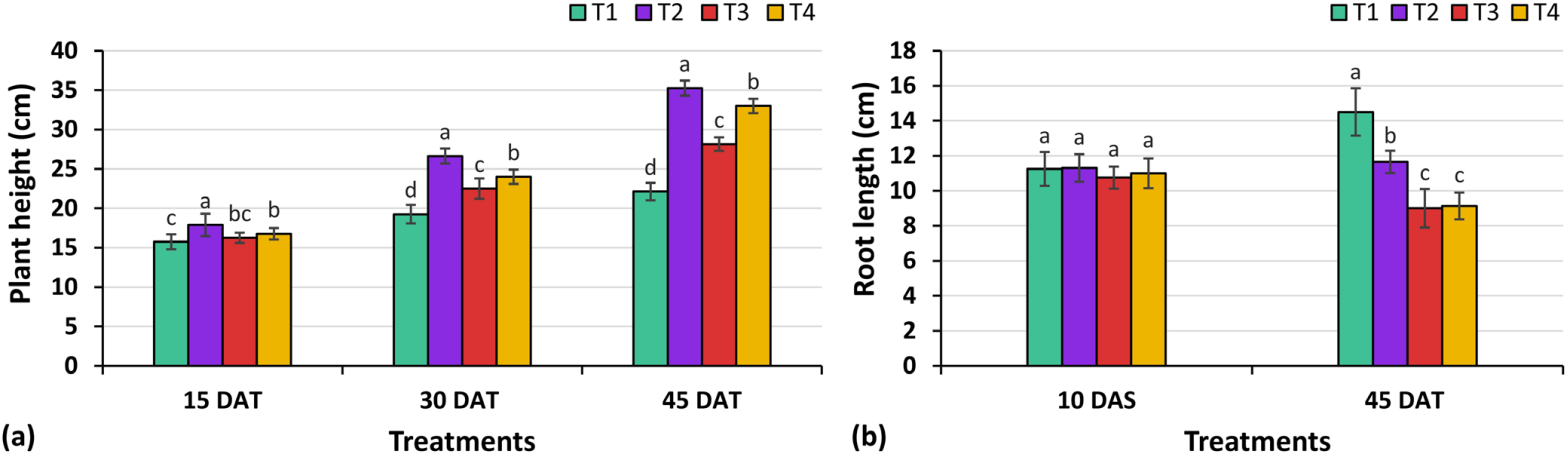
Effect of different treatments on plant height and root length of kangkong. Here, different lowercase letters indicate significant differences (*p* ≤ 0.05). Vertical bars represent the standard error of the mean (*n* = 4).

Plant height is a significant growth‐contributing characteristic of kangkong. Various sources of organic and non‐organic nutrient media notably influenced (*p* ≤ 0.05) the height of kangkong (Figure 6). The tallest kangkong plants were consistently observed in the T2 treatment, with heights of 17.88, 26.63, and 35.25 cm at 15, 30, and 45 days after transplanting (DAT). T4 treatment followed closely with heights of 16.75, 24.00, and 33.00 cm, while T1 treatment produced the shortest plants, measuring 15.75, 19.25, and 22.13 cm at the respective time points. The organic treatment T4 showed improved plant height, comparable to the non‐organic treatment T2, and these results are consistent with the findings of Alneyadi et al. (2024). Regarding root length, no significant differences (*p* > 0.05) were observed among treatments at 10 DAT. However, at 45 DAT, significant differences emerged (*p* ≤ 0.05). The longest root length was recorded in treatment T1 at 14.50 cm, while the shortest was observed in treatment T3 at 9.00 cm. The variation may be attributed to the protective effect of cocopeat, where denser root growth was observed. In contrast, the better root growth in pot culture could be due to variations in development patterns or the physical support provided by the soil. These findings align with Endoh et al. (2024), who observed similar growth patterns in hydroponic lettuce with liquid organic fertilizers.

#### 3.3.4 Fresh and Dry Biomass

Significant differences (*p* ≤ 0.001) were observed in the fresh and dry weights of kangkong shoots, roots, and total plant mass across the different treatments (Table 2). The highest fresh shoot weight was recorded in treatment T2 at 17.60 g, followed closely by T4 at 16.77 g, while T1 had the lowest fresh shoot weight at 6.75 g. For fresh root weight, T2 again showed the highest value at 7.90 g, comparable to T4 at 7.50 g, whereas T1 recorded the lowest weight at 4.08 g. The total fresh weight per plant was highest in T2 at 25.50 g, followed by T4 at 24.27 g, whereas T1 showed the lowest total fresh weight at 10.82 g. In terms of dry weight, the maximum dry shoot weight was observed in T2 at 2.30 g, followed by T4 at 2.19 g, while T1 had the lowest at 0.98 g. The highest dry root weight was recorded in T2 at 1.08 g and T4 at 1.06 g, whereas T1 had the lowest at 0.35 g. Consequently, the total dry weight per plant was highest in T2 at 3.38 g, followed closely by T4 at 3.25 g, while T1 recorded the lowest total dry weight at 1.33 g. These results indicate that non‐organic treatment T2 significantly promoted kangkong growth, while organic treatment T4 showed comparable results. Both treatments demonstrated superior growth compared to T1. These findings are consistent with those of Williams and Nelson (2016) and Ahmed et al. (2021), who reported similar improvements in plant growth with organic nutrient supplementation.

**TABLE 2:** Effect of different treatments on fresh and dry biomass of kangkong.

Treatments	Fresh weight of shoot per plant (g)	Fresh weight of root per plant (g)	Total fresh weight per plant (g)	Dry weight of shoot per plant (g)	Dry weight of root per plant (g)	Total dry weight per plant (g)
T1	06.75 c	4.08 c	10.82 d	0.98 c	0.35 c	1.33 d
T2	17.60 a	7.90 a	25.50 a	2.30 a	1.08 a	3.38 a
T3	14.45 b	6.78 b	21.22 c	1.93 b	0.93 b	2.86 c
T4	16.77 a	7.50 a	24.27 b	2.19 a	1.06 a	3.25 b
Level of sig.	***	***	***	***	***	***
CV (%)	4.58	5.80	2.41	3.72	6.37	2.20
LSD _0.05_	1.018	0.609	0.790	0.113	0.088	0.101

*Note:* Here, the different lowercase letters in a column indicate significant differences among different treatments (*p* ≤ 0.05). Significance level: ‘***’ *p* ≤ 0.001.

#### 3.3.5 Total Yield Per Unit of Plot

**FIGURE 7:**
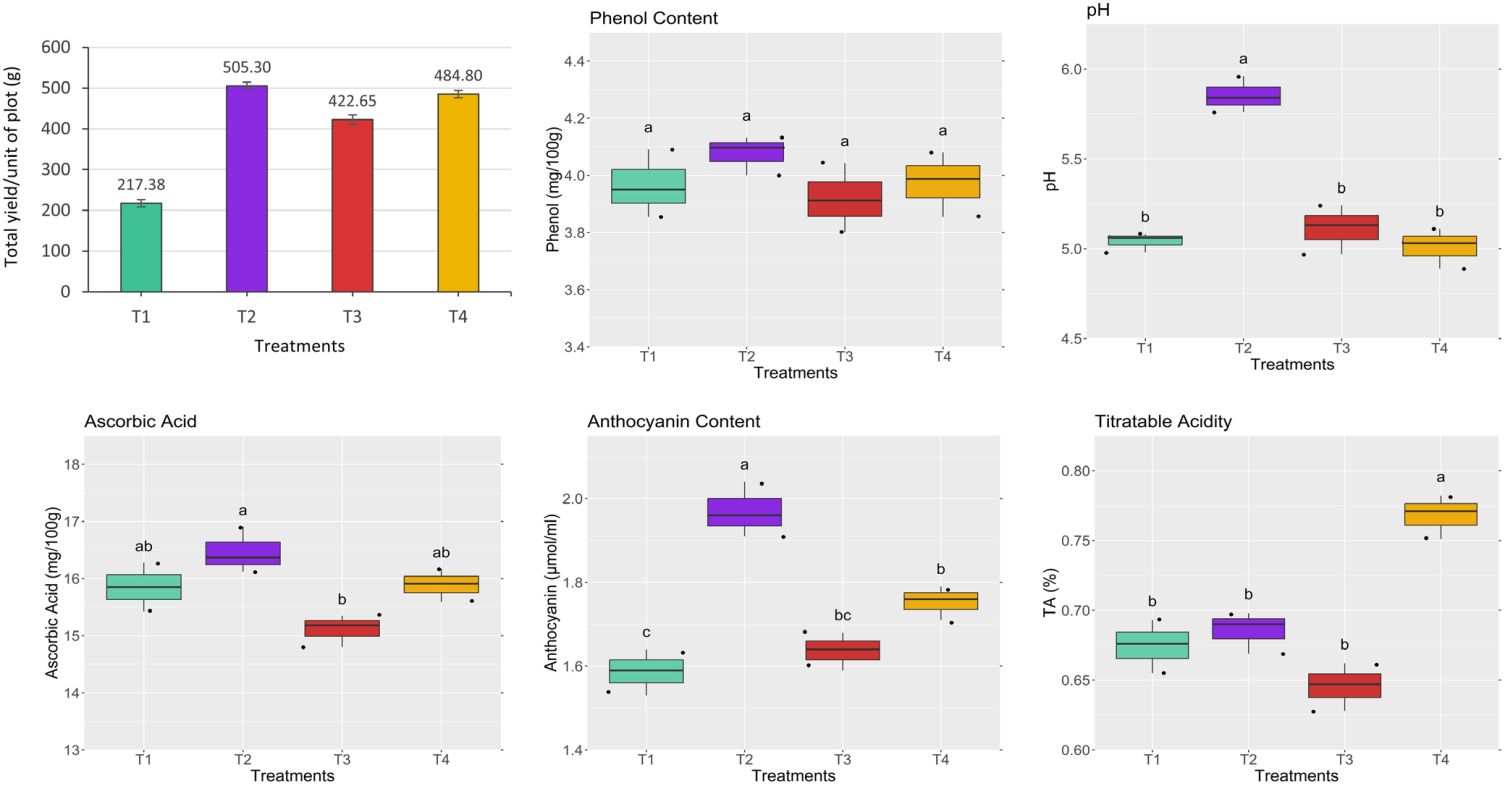
Effect of different treatments on total yield and nutritional quality of kangkong. Here, different lowercase letters indicate significant differences (*p* ≤ 0.05). Vertical bars represent the standard error of the mean (*n* = 3).

The total yield per unit plot of kangkong showed significant variation (*p* ≤ 0.05) across different treatments (Figure 7). The highest yield was observed in treatment T2 at 505.30 g, followed closely by T4 at 484.80 g, whereas the lowest yield of 217.38 g was recorded in T1. The results indicate that the highest yield was achieved with non‐organic treatment T2, which was comparable to the yield obtained with organic treatment T4. These findings suggest that organic treatment T4 effectively sustained plant growth in aeroponic systems. The results are consistent with previous studies by Williams and Nelson (2016), Alneyadi et al. (2024) and Ahmed et al. (2021), all of which reported enhanced plant growth with liquid organic fertilizers.

#### 3.3.6 Physicochemical Properties

Significant variations (*p* ≤ 0.05) were observed in pH, ascorbic acid, anthocyanin content, and titratable acidity (TA) of kangkong across different treatments (Figure 7). However, no significant (*p* > 0.05) difference was found in phenolic content, which ranged from 3.92 to 4.08 mg/100 g Fw. Treatment T2 exhibited the highest pH value at 5.85, while the lowest pH was recorded in treatment T4 at 5.01, which was statistically similar to treatments T1 and T3. The highest ascorbic acid content was observed in treatment T2 at 16.47 mg/100 g Fw, which was statistically comparable to treatments T1 and T4. In contrast, the lowest ascorbic acid content was recorded in treatment T3 at 15.11 mg/100 g Fw. In terms of anthocyanin content, treatment T2 showed the highest value at 1.97 μmol/mL, whereas the lowest was recorded in treatment T1 at 1.59 μmol/mL. Regarding titratable acidity, treatment T4 exhibited the maximum TA at 0.768%, whereas the lowest TA was observed in treatment T3 at 0.646%. The results demonstrate that the organic treatment T4 effectively maintained all measured nutritional qualities, comparable to the non‐organic treatment T2 and pot culture T1. In addition, TA (%) was higher in T4 than in the other treatments, indicating a more robust flavor. The elevated levels of phenolic content, ascorbic acid, anthocyanins, and TA play a key role in enhancing the nutritional value of kangkong (Hossain 2021; Khwankaew et al. 2018). In line with the hypothesis, these findings suggest that organic treatment T4 effectively provided essential nutrients to maintain the nutritional quality, offering a viable alternative to chemical fertilizers for promoting organic food production and sustainable agriculture.

#### 3.3.7 PCA Biplot

**FIGURE 8:**
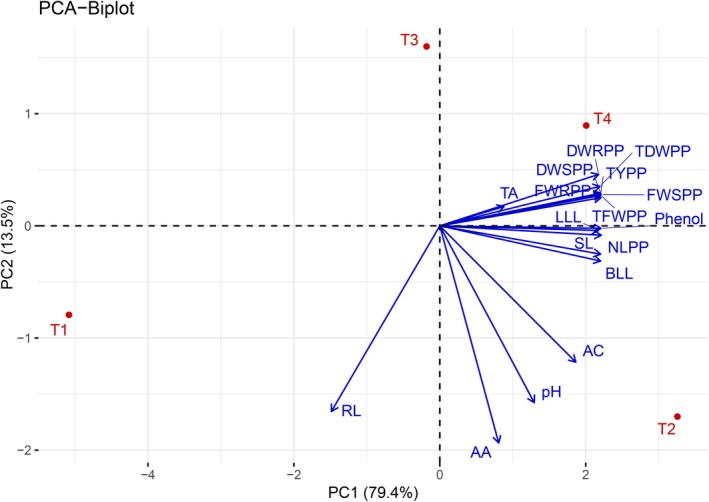
Principal component analysis (PCA) of the different treatments across various morphological and biochemical parameters. Here, AA, Ascorbic acid, AC, Anthocyanin content, BLL, Breadth of largest leaf, DWRPP, Dry weight of root per plant, DWSPP, Dry weight of shoot per plant, FWRPP, Fresh weight of root per plant, FWSPP, Fresh weight of shoot per plant, LLL, Length of largest leaf, NLPP, Number of leaves per plant, RL, Root length, SL, Shoot length, TA, Titratable Acidity, TDWPP, Total dry weight per plant, TFWPP, Total fresh weight per plant, TYPP, Total yield per plot.

Principal Component Analysis (PCA) was performed (Figure 8) to explore the relationships among the measured morphological and biochemical parameters and to determine the overall pattern of variation among the treatments (T1–T4). The first two principal components (PC1 and PC2) accounted for 92.9% of the total variance, with PC1 explaining 79.4% and PC2 contributing 13.5%. PC1 showed a strong positive correlation with the length of the largest leaf (LLL), fresh and dry weights of shoot and root (FWSPP, FWRPP, DWSPP, DWRPP), total fresh and dry weights per plant (TFWPP, TDWPP), titratable acidity (TA), and total yield per plot (TYPP). In contrast, PC2 exhibited a positive association with phenolic content (Phenol), shoot length (SL), number of leaves per plant (NLPP), pH, ascorbic acid (AA), and anthocyanin content (AC). The PCA biplot distinctly separates treatments (T1–T4), revealing clear clustering patterns linked to variations in growth, yield, and biochemical profiles, thereby identifying key traits underpinning treatment‐specific plant performance.

## 4 Conclusion

This study investigates the feasibility of repurposing agrowaste into liquid organic fertilizers for vertical aeroponic systems and reducing nutrient solution temperature fluctuations to provide optimal growth conditions, a crucial step towards sustainable agriculture. Following an anaerobic digestion process, liquid organic fertilizer formulations F6 and F4 emerged as optimal blends, boasting ideal pH levels, EC, and nutrient profiles. Comparative analyses demonstrated that liquid organic fertilizer formulation F4 exhibited comparable growth performance and nutritional quality to chemical nutrients, while offering several advantages, including reduced production costs, enhanced sustainability, and lower environmental impact through the valorization of agrowaste and minimized chemical use. Furthermore, the incorporation of a 1.5‐in. silty clay soil layer in the vertical aeroponic system effectively stabilized temperature fluctuations, providing an optimal environment for cultivation. The integration of this silty clay soil layer into commercial vertical aeroponic systems could support climate‐smart agriculture by alleviating the adverse effects of extreme temperatures, whether hot or cold. Future research should focus on incorporating effective microorganisms into liquid organic fertilizers to accelerate the fermentation process and evaluate the fertilizer's impact on a wider range of vegetables to fully assess its potential.

## Funding

The authors have nothing to report.

## Conflicts of Interest

The authors declare no conflicts of interest.

## Supporting information


**Figure S1:** Flowchart of liquid organic fertilizer production through anaerobic digestion.
**Figure S2:** Temperature stabilization of nutrient solution in vertical aeroponic systems.
**Figure S3:** Schematic representation of seedling pot preparation and complete vertical aeroponic systems.
**Figure S4:** Flowchart of the experimental design.
**Table S1:** Characterization of chemical nutrients solution A and B: pH, EC, and nutrient content (N, P, K).
**Appendix S1:** ANOVA for pH of liquid organic fertilizers.
**Appendix S2:** ANOVA for Electrical conductivity (EC) of liquid organic fertilizers.
**Appendix S3:** ANOVA for total nitrogen (N) of liquid organic fertilizers.
**Appendix S4:** ANOVA for available phosphorus (P) of liquid organic fertilizers.
**Appendix S5:** ANOVA for exchangeable potassium (K) of liquid organic fertilizers.
**Appendix S6:** ANOVA for the number of leaves per plant of kangkong.
**Appendix S7:** ANOVA for the length (cm) of the largest leaf of kangkong.
**Appendix S8:** ANOVA for the breadth (cm) of the largest leaf of kangkong.
**Appendix S9:** ANOVA for plant height (cm) of kangkong.
**Appendix S10:** ANOVA for length of root.
**Appendix S11:** ANOVA for fresh weight of shoot per plant of kangkong.
**Appendix S12:** ANOVA for fresh weight of root per plant of kangkong.
**Appendix S13:** ANOVA for total fresh weight per plant of kangkong.
**Appendix S14:** ANOVA for dry weight of shoot per plant of kangkong.
**Appendix S15:** ANOVA for dry weight of root per plant of kangkong.
**Appendix S16:** ANOVA for total dry weight per plant.
**Appendix S17:** ANOVA for total yield of kangkong.
**Appendix S18:** ANOVA for phenol content of kangkong.
**Appendix S19:** ANOVA for pH of kangkong.
**Appendix S20:** ANOVA for ascorbic acid content of kangkong.
**Appendix S21:** ANOVA for anthocyanin content of kangkong.
**Appendix S22:** ANOVA for titratable acidity (TA) of kangkong.

## Data Availability

The data that supports the findings of this study are available in the Supporting Information of this article.
