# Supplementary material for: Agrowaste‐Derived Liquid Organic Fertilizer and Temperature Stabilization in Vertical Aeroponics for Crop Production
Source: Plant Environ Interact. 2026 Apr 27;7(3):e70153. doi: 10.1002/pei3.70153 (PMC13112419; doi:10.1002/pei3.70153)
Supplement: Supplementary file 1 — Figure S1: Flowchart of liquid organic fertilizer production through anaerobic digestion. Figure S2: Temperature stabilization of nutrient solution in vertical aeroponic systems. Figure S3: Schematic representation of seedling pot preparation and complete vertical aeroponic systems. Figure S4: Flowchart of the experimental design. Table S1: Characterization of chemical nutrients solution A and B: pH, EC, and nutrient content (N, P, K). Appendix S1: ANOVA for pH of liquid organic fertilizers. Appendix S2: ANOVA for Electrical conductivity (EC) of liquid organic fertilizers. Appendix S3: ANOVA for total nitrogen (N) of liquid organic fertilizers. Appendix S4: ANOVA for available phosphorus (P) of liquid organic fertilizers. Appendix S5: ANOVA for exchangeable potassium (K) of liquid organic fertilizers. Appendix S6: ANOVA for the number of leaves per plant of kangkong. Appendix S7: ANOVA for the length (cm) of the largest leaf of kangkong. Appendix S8: ANOVA for the breadth (cm) of the largest leaf of kangkong. Appendix S9: ANOVA for plant height (cm) of kangkong. Appendix S10: ANOVA for length of root. Appendix S11: ANOVA for fresh weight of shoot per plant of kangkong. Appendix S12: ANOVA for fresh weight of root per plant of kangkong. Appendix S13: ANOVA for total fresh weight per plant of kangkong. Appendix S14: ANOVA for dry weight of shoot per plant of kangkong. Appendix S15: ANOVA for dry weight of root per plant of kangkong. Appendix S16: ANOVA for total dry weight per plant. Appendix S17: ANOVA for total yield of kangkong. Appendix S18: ANOVA for phenol content of kangkong. Appendix S19: ANOVA for pH of kangkong. Appendix S20: ANOVA for ascorbic acid content of kangkong. Appendix S21: ANOVA for anthocyanin content of kangkong. Appendix S22: ANOVA for titratable acidity (TA) of kangkong. [file PEI3-7-e70153-s001.docx]

**Supplementary Materials:**


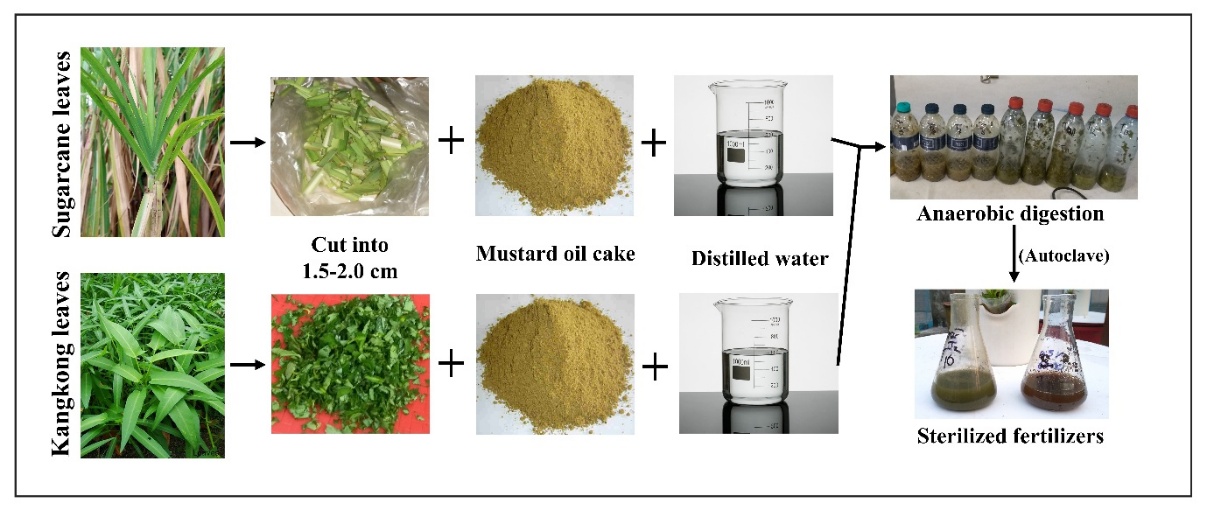


**Figure S1:** Flowchart of liquid organic fertilizer production through anaerobic digestion.


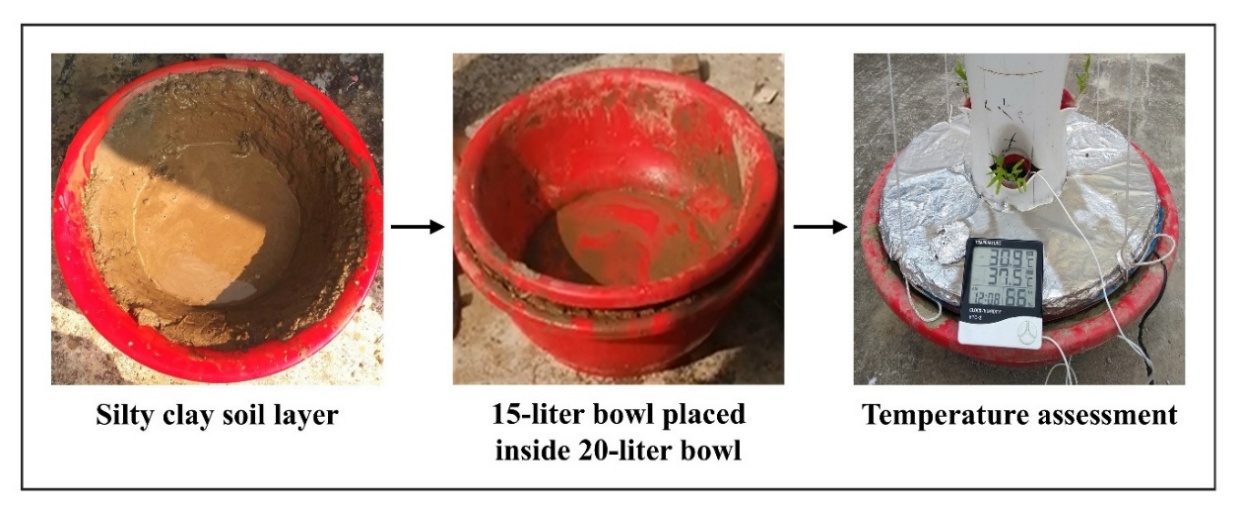


**Figure S2:** Temperature stabilization of nutrient solution in vertical aeroponic systems.


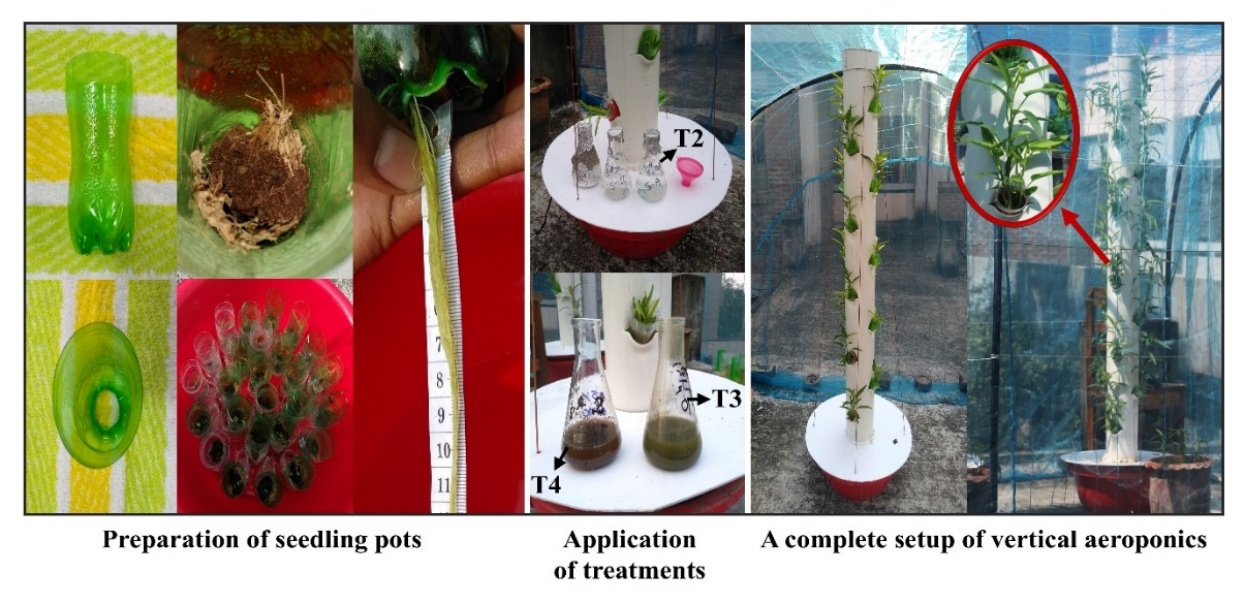


**Figure S3:** Schematic representation of seedling pot preparation and complete vertical aeroponic systems.


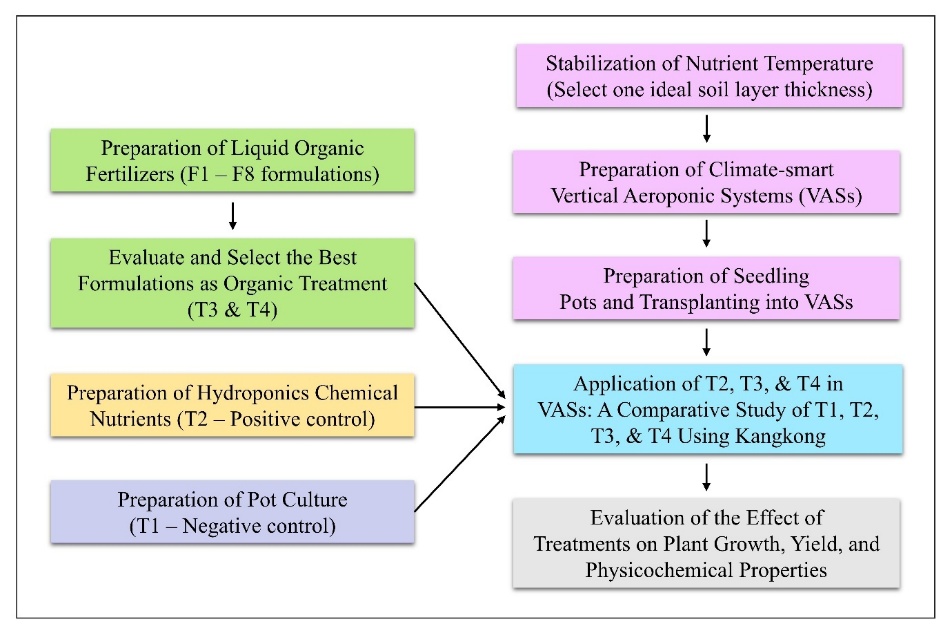


**Figure S4:** Flowchart of the experimental design.

**Table S1.** Characterization of chemical nutrients solution A and B: pH, EC, and nutrient content (N, P, K).

| Chemical Nutrients | pH | EC (dS/m) | Total N (%) | Total P (%) | Total K (%) |
| --- | --- | --- | --- | --- | --- |
| A | 6.53 | 2.89 | 0.126 | 0.008 | 0.004 |
| B | 6.17 | 3.08 | 0.179 | 0.257 | 2.125 |

**Appendices:**

**Appendix 1: ANOVA for pH of liquid organic fertilizers.**

| **Days** | **Source** | **Degrees of Freedom** | **Sum of Squares** | **Mean Square** | **F value** | **Probability** |
| --- | --- | --- | --- | --- | --- | --- |
| 15 DAF | Treatment | 7 | 27.0759 | 3.8679 | 126.75 | 0.0000 |
|  | Error | 16 | 0.4883 | 0.0305 |  |  |
|  | Total | 23 | 27.5642 | 3.8984 |  |  |
| 30 DAF | Treatment | 7 | 45.0000 | 6.4285 | 183.67 | 0.0000 |
|  | Error | 16 | 0.5600 | 0.0350 |  |  |
|  | Total | 23 | 45.5600 | 6.4635 |  |  |

**Appendix 2: ANOVA for Electrical conductivity (EC) of liquid organic fertilizers.**

| **Days** | **Source** | **Degrees of Freedom** | **Sum of Squares** | **Mean Square** | **F value** | **Probability** |
| --- | --- | --- | --- | --- | --- | --- |
| 15 DAF | Treatment | 7 | 143.618 | 20.5169 | 863.42 | 0.0000 |
|  | Error | 16 | 0.380 | 0.0238 |  |  |
|  | Total | 23 | 143.998 | 20.5407 |  |  |
| 30 DAF | Treatment | 7 | 222.944 | 31.8492 | 636.98 | 0.0000 |
|  | Error | 16 | 0.800 | 0.0500 |  |  |
|  | Total | 23 | 223.744 | 31.8992 |  |  |

**Appendix 3: ANOVA for total nitrogen (N) of liquid organic fertilizers.**

| **Days** | **Source** | **Degrees of Freedom** | **Sum of Squares** | **Mean Square** | **F value** | **Probability** |
| --- | --- | --- | --- | --- | --- | --- |
| 15 DAF | Treatment | 7 | 0.05573 | 7.961E-03 | 51.64 | 0.0000 |
|  | Error | 16 | 0.00247 | 1.542E-04 |  |  |
|  | Total | 23 | 0.05820 | 0.008 |  |  |
| 30 DAF | Treatment | 7 | 0.05100 | 7.286E-03 | 41.63 | 0.0000 |
|  | Error | 16 | 0.00280 | 1.750E-04 |  |  |
|  | Total | 23 | 0.05380 | 0.015 |  |  |

**Appendix 4: ANOVA for available phosphorus (P) of liquid organic fertilizers.**

| **Days** | **Source** | **Degrees of Freedom** | **Sum of Squares** | **Mean Square** | **F value** | **Probability** |
| --- | --- | --- | --- | --- | --- | --- |
| 15 DAF | Treatment | 7 | 3.363E-04 | 4.804E-04 | 33.91 | 0.0000 |
|  | Error | 16 | 2.267E-05 | 1.417E-06 |  |  |
|  | Total | 23 | 3.590E-04 | 6.221E-10 |  |  |
| 30 DAF | Treatment | 7 | 4.886E-04 | 6.980E-05 | 51.95 | 0.0000 |
|  | Error | 16 | 2.150E-05 | 1.344E-06 |  |  |
|  | Total | 23 | 5.101E-04 | 8.324E-11 |  |  |

**Appendix 5: ANOVA for exchangeable potassium (K) of liquid organic fertilizers.**

| **Days** | **Source** | **Degrees of Freedom** | **Sum of Squares** | **Mean Square** | **F value** | **Probability** |
| --- | --- | --- | --- | --- | --- | --- |
| 15 DAF | Treatment | 7 | 0.58958 | 0.08423 | 253.47 | 0.0000 |
|  | Error | 16 | 0.00532 | 0.00033 |  |  |
|  | Total | 23 | 0.59490 | 0.08456 |  |  |
| 30 DAF | Treatment | 7 | 0.70424 | 0.10061 | 19.31 | 0.0000 |
|  | Error | 16 | 0.08335 | 0.00521 |  |  |
|  | Total | 23 | 0.78759 | 0.10582 |  |  |

**Appendix 6: ANOVA for the number of leaves per plant of kangkong.**

| **Days** | **Source** | **Degrees of Freedom** | **Sum of Squares** | **Mean Square** | **F value** | **Probability** |
| --- | --- | --- | --- | --- | --- | --- |
|  | Replication | 3 | 12.250 | 4.083 | 7.0000 | 0.0100 |
| 15 DAT | Treatment | 3 | 20.250 | 6.750 | 11.5714 | 0.0019 |
|  | Error | 9 | 5.250 | 0.583 |  |  |
|  | Total | 15 | 37.750 |  |  |  |
|  | Replication | 3 | 16.500 | 5.500 | 19.8000 | 0.0003 |
| 30 DAT | Treatment | 3 | 29.000 | 9.667 | 34.8000 | 0.0000 |
|  | Error | 9 | 2.500 | 0.278 |  |  |
|  | Total | 15 | 48.000 |  |  |  |
|  | Replication | 3 | 5.688 | 1.896 | 1.414 | 0.3012 |
| 45 DAT | Treatment | 3 | 186.188 | 62.063 | 46.3057 | 0.0000 |
|  | Error | 9 | 12.063 | 1.340 |  |  |
|  | Total | 15 | 203.938 |  |  |  |

**Appendix 7: ANOVA for the length (cm) of the largest leaf of kangkong.**

| **Days** | **Source** | **Degrees of Freedom** | **Sum of Squares** | **Mean Square** | **F value** | **Probability** |
| --- | --- | --- | --- | --- | --- | --- |
|  | Replication | 3 | 2.477 | 0.826 | 4.4881 | 0.0346 |
| 15DAT | Treatment | 3 | 11.852 | 3.951 | 21.4756 | 0.0002 |
|  | Error | 9 | 1.656 | 0.184 |  |  |
|  | Total | 15 | 15.984 |  |  |  |
|  | Replication | 3 | 2.055 | 0.685 | 7.3393 | 0.0086 |
| 30DAT | Treatment | 3 | 14.405 | 4.802 | 51.4464 | 0.0000 |
|  | Error | 9 | 0.840 | 0.093 |  |  |
|  | Total | 15 | 17.300 |  |  |  |
|  | Replication | 3 | 1.188 | 0.396 | 3.0000 | 0.0877 |
| 45DAT | Treatment | 3 | 27.063 | 9.021 | 68.3684 | 0.0000 |
|  | Error | 9 | 1.188 | 0.132 |  |  |
|  | Total | 15 | 29.438 |  |  |  |

**Appendix 8: ANOVA for the breadth (cm) of the largest leaf of kangkong.**

| **Days** | **Source** | **Degrees of Freedom** | **Sum of Squares** | **Mean Square** | **F value** | **Probability** |
| --- | --- | --- | --- | --- | --- | --- |
|  | Replication | 3 | 0.057 | 0.019 | 10.9200 | 0.0024 |
| 15DAT | Treatment | 3 | 0.057 | 0.019 | 10.9200 | 0.0024 |
|  | Error | 9 | 0.016 | 0.002 |  |  |
|  | Total | 15 | 0.129 |  |  |  |
|  | Replication | 3 | 0.035 | 0.012 | 3.5000 | 0.0628 |
| 30DAT | Treatment | 3 | 0.555 | 0.185 | 55.5000 | 0.0000 |
|  | Error | 9 | 0.030 | 0.003 |  |  |
|  | Total | 15 | 0.620 |  |  |  |
|  | Replication | 3 | 0.022 | 0.007 | 0.7664 |  |
| 45DAT | Treatment | 3 | 0.797 | 0.266 | 27.9197 | 0.0001 |
|  | Error | 9 | 0.086 | 0.010 |  |  |
|  | Total | 15 | 0.904 |  |  |  |

**Appendix 9: ANOVA for plant height (cm) of kangkong.**

| **Days** | **Source** | **Degrees of Freedom** | **Sum of Squares** | **Mean Square** | **F value** | **Probability** |
| --- | --- | --- | --- | --- | --- | --- |
|  | Replication | 3 | 7.922 | 2.642 | 7.8808 | 0.0069 |
| 15 DAT | Treatment | 3 | 9.922 | 3.307 | 9.8705 | 0.0033 |
|  | Error | 9 | 3.016 | 0.335 |  |  |
|  | Total | 15 | 20.859 |  |  |  |
|  | Replication | 3 | 4.922 | 1.641 | 4.2000 | 0.0408 |
| 30 DAT | Treatment | 3 | 113.672 | 37.891 | 97.0000 | 0.0000 |
|  | Error | 9 | 3.516 | 0.391 |  |  |
|  | Total | 15 | 122.109 |  |  |  |
|  | Replication | 3 | 7.250 | 2.417 | 2.4507 | 0.1302 |
| 45 DAT | Treatment | 3 | 406.125 | 135.375 | 137.2817 | 0.0000 |
|  | Error | 9 | 8.875 | 0.986 |  |  |
|  | Total | 15 | 422.250 |  |  |  |

**Appendix 10: ANOVA for length of root.**

| **Days** | **Source** | **Degrees of Freedom** | **Sum of Squares** | **Mean Square** | **F value** | **Probability** |
| --- | --- | --- | --- | --- | --- | --- |
|  | Replication | 3 | 0.172 | 0.057 | 0.0857 |  |
| 10 DDT | Treatment | 3 | 0.922 | 0.307 | 0.4597 |  |
|  | Error | 9 | 6.016 | 0.668 |  |  |
|  | Total | 15 | 7.109 |  |  |  |
|  | Replication | 3 | 0.727 | 0.242 | 0.2469 |  |
| 45DAT | Treatment | 3 | 80.677 | 26.892 | 27.4081 | 0.0001 |
|  | Error | 9 | 8.831 | 0.981 |  |  |
|  | Total | 15 | 90.234 |  |  |  |

**Appendix 11: ANOVA for fresh weight of shoot per plant of kangkong.**

| **Source** | **Degrees of Freedom** | **Sum of Squares** | **Mean Square** | **F value** | **Probability** |
| --- | --- | --- | --- | --- | --- |
| Replication | 3 | 2.282 | 0.761 | 1.8778 | 0.2038 |
| Treatment | 3 | 293.522 | 97.841 | 241.5405 | 0.0000 |
| Error | 9 | 3.646 | 0.405 |  |  |
| Total | 15 | 299.449 |  |  |  |

**Appendix 12: ANOVA for fresh weight of root per plant of kangkong.**

| **Source** | **Degrees of Freedom** | **Sum of Squares** | **Mean Square** | **F value** | **Probability** |
| --- | --- | --- | --- | --- | --- |
| Replication | 3 | 0.272 | 0.091 | 0.6276 |  |
| Treatment | 3 | 35.603 | 11.868 | 82.0019 | 0.0000 |
| Error | 9 | 1.303 | 0.145 |  |  |
| Total | 15 | 37.178 | 12.013 |  |  |

**Appendix 13: ANOVA for total fresh weight per plant of kangkong.**

| **Source** | **Degrees of Freedom** | **Sum of Squares** | **Mean Square** | **F value** | **Probability** |
| --- | --- | --- | --- | --- | --- |
| Replication | 3 | 2.927 | 0.976 | 3.9991 | 0.0460 |
| Treatment | 3 | 533.497 | 177.832 | 728.9454 | 0.0000 |
| Error | 9 | 2.196 | 0.244 |  |  |
| Total | 15 | 538.619 |  |  |  |

**Appendix 14: ANOVA for dry weight of shoot per plant of kangkong.**

| **Source** | **Degrees of Freedom** | **Sum of Squares** | **Mean Square** | **F value** | **Probability** |
| --- | --- | --- | --- | --- | --- |
| Replication | 3 | 0.020 | 0.007 | 1.4131 | 0.3016 |
| Treatment | 3 | 4.346 | 1.449 | 305.2707 | 0.0000 |
| Error | 9 | 0.043 | 0.005 |  |  |
| Total | 15 | 4.409 |  |  |  |

**Appendix 15: ANOVA for dry weight of root per plant of kangkong.**

| **Source** | **Degrees of Freedom** | **Sum of Squares** | **Mean Square** | **F value** | **Probability** |
| --- | --- | --- | --- | --- | --- |
| Replication | 3 | 0.065 | 0.022 | 7.3655 | 0.0085 |
| Treatment | 3 | 1.391 | 0.464 | 156.7669 | 0.0000 |
| Error | 9 | 0.027 | 0.003 |  |  |
| Total | 15 | 1.483 |  |  |  |

**Appendix 16: ANOVA for total dry weight per plant**

| **Source** | **Degrees of Freedom** | **Sum of Squares** | **Mean Square** | **F value** | **Probability** |
| --- | --- | --- | --- | --- | --- |
| Replication | 3 | 0.037 | 0.012 | 3.4917 | 0.0632 |
| Treatment | 3 | 10.642 | 3.547 | 1003.7390 | 0.0000 |
| Error | 9 | 0.032 | 0.004 |  |  |
| Total | 15 | 10.711 |  |  |  |

**Appendix 17: ANOVA for total yield of kangkong.**

| **Source** | **Degrees of Freedom** | **Sum of Squares** | **Mean Square** | **F value** | **Probability** |
| --- | --- | --- | --- | --- | --- |
| Replication | 3 | 1.204 | 0.401 | 0.401 | 0.1896 |
| Treatment | 3 | 13073.153 | 4357.718 | 2059.5214 | 0.0000 |
| Error | 9 | 19.043 | 2.116 |  |  |
| Total | 15 | 13093.400 |  |  |  |

**Appendix 18: ANOVA for phenol content of kangkong.**

| **Source** | **Degrees of Freedom** | **Sum of Squares** | **Mean Square** | **F value** | **Probability** |
| --- | --- | --- | --- | --- | --- |
| Replication | 1 | 0.035778 | 0.035778 | 4.4461 | 0.07294 |
| Treatment | 3 | 0.039762 | 0.013254 | 1.6470 | 0.26374 |
| Error | 7 | 0.056330 | 0.008047 |  |  |
| Total | 11 | 0.13187 |  |  |  |

**Appendix 19: ANOVA for pH of kangkong.**

| **Source** | **Degrees of Freedom** | **Sum of Squares** | **Mean Square** | **F value** | **Probability** |
| --- | --- | --- | --- | --- | --- |
| Replication | 1 | 0.02761 | 0.02761 | 3.2257 | 0.1156 |
| Treatment | 3 | 1.45296 | 0.48432 | 56.5786 | 2.834E-05 |
| Error | 7 | 0.05992 | 0.00856 |  |  |
| Total | 11 | 1.54049 |  |  |  |

**Appendix 20: ANOVA for ascorbic acid content of kangkong.**

| **Source** | **Degrees of Freedom** | **Sum of Squares** | **Mean Square** | **F value** | **Probability** |
| --- | --- | --- | --- | --- | --- |
| Replication | 1 | 0.00101 | 0.00101 | 0.0069 | 0.9360 |
| Treatment | 3 | 2.78323 | 0.92774 | 6.3528 | 0.0208 |
| Error | 7 | 1.02225 | 0.14604 |  |  |
| Total | 11 | 3.80649 |  |  |  |

**Appendix 21: ANOVA for anthocyanin content of kangkong.**

| **Source** | **Degrees of Freedom** | **Sum of Squares** | **Mean Square** | **F value** | **Probability** |
| --- | --- | --- | --- | --- | --- |
| Replication | 1 | 0.007812 | 0.007812 | 3.8546 | 0.09038 |
| Treatment | 3 | 0.261667 | 0.087222 | 43.0348 | 7.04E-05 |
| Error | 7 | 0.014187 | 0.002027 |  |  |
| Total | 11 | 0.283666 |  |  |  |

**Appendix 22: ANOVA for titratable acidity (TA) of kangkong.**

| **Source** | **Degrees of Freedom** | **Sum of Squares** | **Mean Square** | **F value** | **Probability** |
| --- | --- | --- | --- | --- | --- |
| Replication | 1 | 2.11E-05 | 2.11E-05 | 0.0664 | 0.804057 |
| Treatment | 3 | 0.024763 | 0.008254 | 25.9468 | 0.000363 |
| Error | 7 | 0.002227 | 0.000318 |  |  |
| Total | 11 | 0.027011 |  |  |  |
